# Spontaneous, sub-acute right lung torsion: a case report

**DOI:** 10.1007/s12055-024-01796-5

**Published:** 2024-08-12

**Authors:** Calixte de La Bourdonnaye, Marion Mauduit, Simon Rouze, Bertrand Richard de Latour

**Affiliations:** Department of Cardiothoracic Surgery, University Hospital of Rennes Pontchaillou, 2 rue Henri le Guilloux, Rennes, 35000 France

**Keywords:** Lobar torsion, Spontaneous lung torsion, General thoracic surgery, Lobectomy, Thoracic CT scan

## Abstract

Lung volvulus is a rare occurrence and is most commonly seen after thoracic surgery or trauma. They are generally associated with a long, thin hilum, with no parenchymal bridge between the lobes. In non-postoperative situations, pleural effusion or pneumothorax would appear to be mandatory. Spontaneous volvuli are not described, especially sub-acutely. We report the case of a patient with an apparently spontaneous lung volvulus. He presented with long prodromal symptoms of haemoptysis and increasing cough. The computed tomography scan showed a complete volvulus of the right lung with signs of non-perfusion of the upper and middle lobes. The patient was successfully treated with volvulus reduction and bi-lobectomy. Torsion is classically known to thoracic surgeons, but is rarely encountered by other specialists. We describe here a sub-acute lung volvulus, apparently spontaneous, easily treated by a simple surgical procedure.

## Introduction

Lung volvulus is a very rare occurrence, particularly known after thoracic surgery. Other aetiologies have been described, notably post-traumatic or associated with neoplasia [1]. The basic anatomical features are a thin, long hilum with no parenchymal bridge between the lobes [2]. In the non-postoperative situation, pleural effusion or pneumothorax seems to be mandatory [3].

We present here the case of a patient who had an apparent spontaneous lung volvulus. We describe the clinical features, para-clinical signs, and a simple and effective surgical treatment for this condition.

## Case report

A 63-year-old man was initially admitted to the hospital with haemoptysis and right lower chest pain. His medical history included seasonal asthma and moderate smoking. He described coughing for 6 months, rarely associated with small haemoptysis. He had consulted his doctor twice. The latter prescribed beta-2-mimetics with aerosolised corticosteroids, followed by oral corticosteroids. After 48 h of oral corticosteroids, the patient presented with increasing right lower chest pain, accentuated by deep inspiration and haemoptysis. He was transferred to a peripheral emergency department. He was stable, with a fever of 38.5 °C and an isolated abolition of the right respiratory sound. The electrocardiogram was normal, and the blood count showed a significant inflammatory syndrome and hypoxaemia (PaO_2_, 62.4 mmHg). On computerized tomography scan (CT scan), the right lobe was described as ‘distended’ with signs of pneumonia due to an obstruction in the intermediate trunk of the right bronchus, with no adenopathy.

**Fig. 1:**
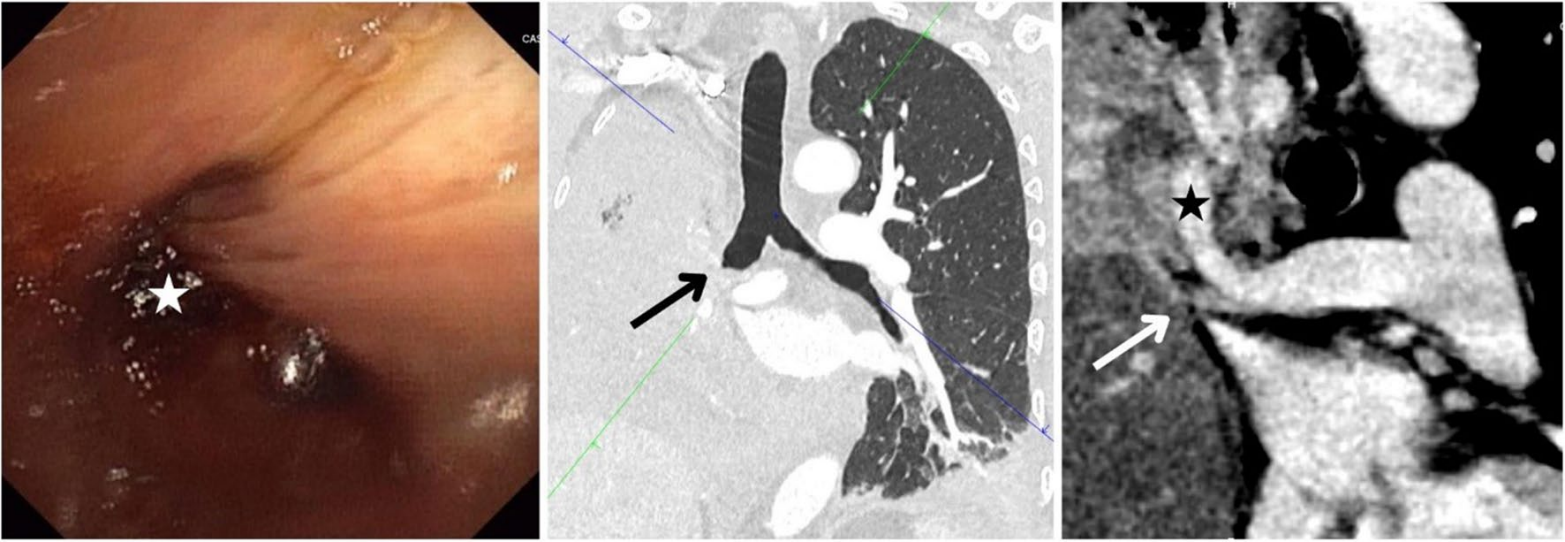
Bronchoscopy and computed tomography (CT) scan showing the hilar torsion. Bronchoscopy shows impenetrable obstruction of the right main bronchus (white star) with active bleeding (appearance of old blood). CT scan shows a sudden obstruction of the right main bronchus (black arrow). The CT scan shows a rotated artery (white arrow), the inferior pulmonary artery (black star) remains patent in the upper position, and the middle and superior pulmonary arteries are narrowed and beside the plane of this image

The patient was initially treated with spiramycin and cefotaxime. After 4 days, the patient had not recovered and blood tests revealed an increased inflammatory syndrome and anaemia (−4 haemoglobin points). On a repeat CT scan, the right lobe was full of ground-glass opacity and the right main bronchus was obstructed with a slight pleural effusion. Bronchoscopy showed impenetrable obstruction of the right main bronchus with active bleeding (Fig. 1). He was transferred to our tertiary hospital centre in pneumology.

**Fig. 2:**
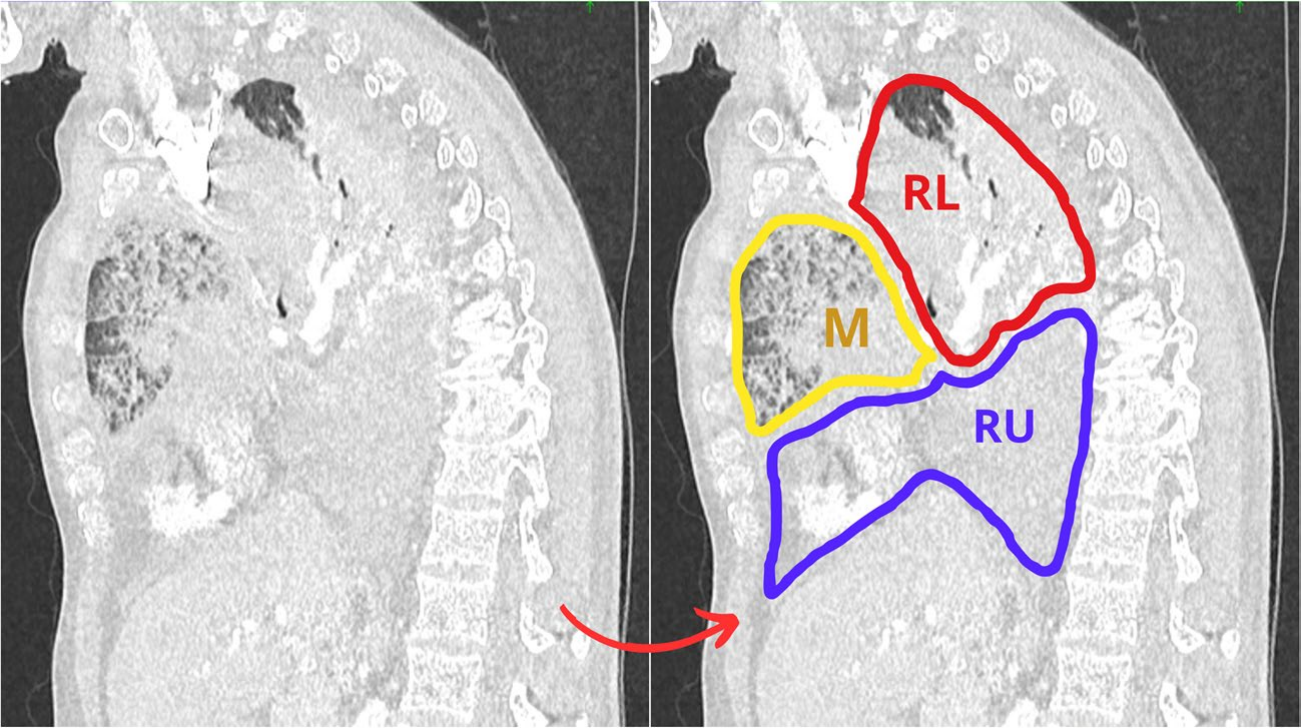
Preoperative computed tomography (CT) scan. The right upper (RU) lobe is in a lower position and completely necrotic (condensation, and haemorrhagic transformation). The middle (M) lobe is also necrotic in an anterior position (ground glass opacities). Both did not have any venous drainage or aeration. The right lower (RL) lobe is in an upper position with ventilated parenchyma remaining in the apex and effective venous drainage

He was haemodynamically stable on 2.5 l of oxygen. Right-sided breath sounds were absent, and there was impaired resonance on the percussion of the chest with decreased transmission of voice sound. A repeat scan showed a 180° torsion of the right lung with extensive necrosis of the right upper and middle lobes. The right upper and middle lobes were not drained due to occlusion of their veins. All arteries in the right lung were patent (lower pulmonary artery was almost not narrowed). Only the right lower lobe did not have an obstructed bronchus (Fig. 2).

**Fig. 3:**
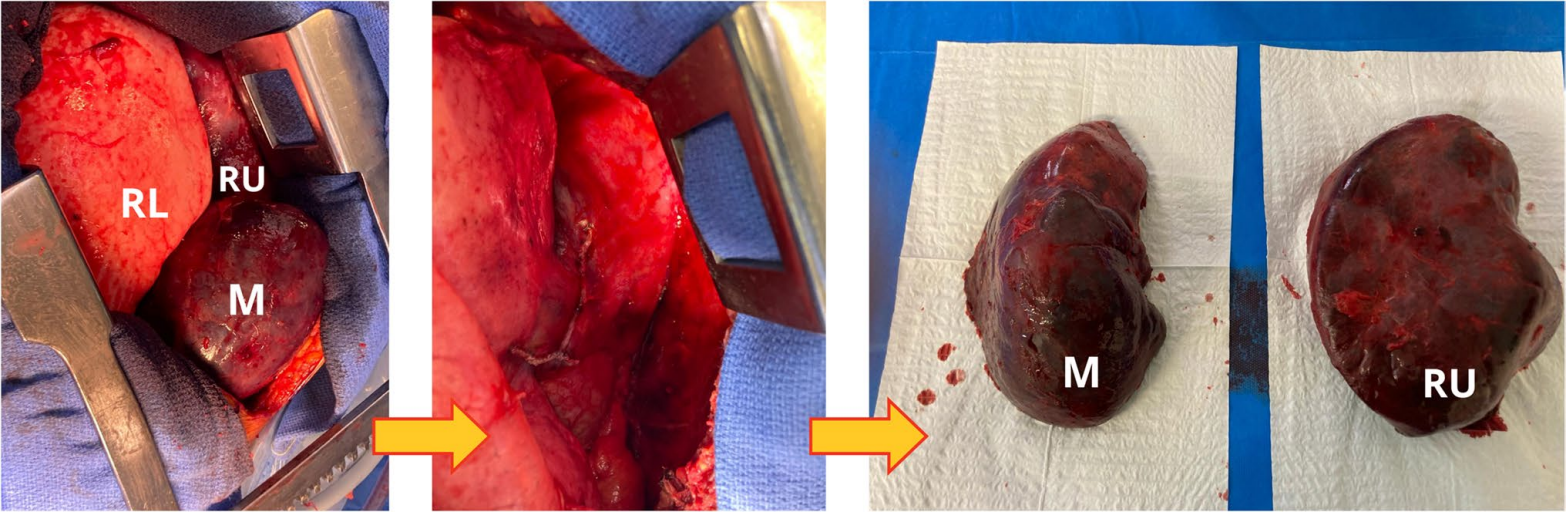
Preoperative images. Necrotic right upper (RU) and middle (M) lobes and well-perfused right lower (RL) lobe. After middle and superior lobectomies (2 automatics staples). The middle and inferior necrotic lobes

The patient was immediately taken to the operating theatre. A pleural effusion consisting of clots was evacuated. The three lobes were rotated back into place by 180°. The right upper and middle lobes were hepatised and hard to palpate. The two pedicles of these necrotic lobes were elongated and very thin, with no parenchymal bridge. A single 45-mm automated staple was used for each pedicle to perform the lobar resection (Fig. 3). The pleural cavity was carefully washed with 6 l of warm water, and the chest was closed.

The patient remained in the intensive care unit for 2 days and then returned to the surgical ward. Chest tubes were removed on days 5 and 6. The patient went home 8 days after the operation. Before he went home, bronchoscopy showed successful healing of the right bronchus and CT scan showed a well-ventilated right lower lobe with signs of allergic aspergillosis (irregular, spontaneously dense bronchocele). The patient was treated with long-term itraconazol (antifungal therapy) due to positive antigenuria for *Aspergillus*.

After 1-year follow-up, the patient is doing well and has returned to a normal daily life.

## Discussion

We have described spontaneous torsion of the right lung. Lobar or pulmonary torsion is a rotation of the lung around its hilum (clockwise or anticlockwise). Contention of the lung is maintained by the hilum with the triangular ligament and the ‘vacuum’ in the pleura maintaining congruence between the shape of the inflated lung and the chest wall.

The risk factors are well defined by the following: long, thin hilum, absence of triangular ligament, absence of parenchymal bridge between lobes, perfect scissure (anatomical risk factor) [2], and pneumothorax or haemothorax (pathological risk factor) [3]. The most common aetiology is surgery (75%), followed by thoracic trauma (25%) [1]. Other possibilities have been described [4, 5], but ‘spontaneous’ torsion is not known to our knowledge.

Herein, several hypotheses can be propounded: the patient suffered from asthma and had *Aspergillus* positive testing, which are probably not associated with lung torsion, but perhaps the prolonged cough effort is a risk factor. He practiced yoga, but it is not known if this activity predisposed to lung torsion.

The clinical presentation of pulmonary torsion is not very specific (cough, haemoptysis, fever, chest pain, etc.) [6, 7]. Auscultation shows a silent chest. In this case, it is difficult to say whether the torsion of the lobe was purely acute or whether there was an earlier sub-torsion that gave rise to earlier symptoms. Usually, this presents as a very acute disease with severe illness within a few hours of torsion. Some torsions, less than 90°, have been seen to regress spontaneously postoperatively, with a less impressive presentation [8].

In general, there is a high degree of inflammation and hypoxia in the blood gases. If the circulation is completely obstructed, hypoxia may be delayed or absent (no flow, no shunt) [7]. The chest X-ray will show classic mobile condensation, changing position as the patient’s position changes.

The diagnosis is determined by bronchoscopy or CT scan [4, 6, 7]. Bronchoscopy shows an impenetrable torsion of the bronchus, sometimes with the passage of blood into the obstruction. On CT scan, there may be condensation with a bronchial-vessel obstruction. Sometimes there is trapped air. It is important to assess the presence of thrombus in the pulmonary vein prior to surgery (see below) [4].

Although medical treatment has been described (rotation < 90°, pauci-symptomatic), the main solution is surgery [1–10]. The anatomical risk factor and the torsion of the hilum often make this surgery easier than many lobectomies: there is little dissection (in our case, only one staple was used per lobe). If the lung is still pink, detorsion preserving the lobe or lung is possible [9]. There are two risks to be aware of. Firstly, cerebrovascular accidents: before detorsion, the pulmonary vein must be clamped intrapericardially (the vessels must be opened proximally) [4, 5]. Even in the case of pulmonary resection, the surgeon must clamp the pulmonary vein. Secondly, systemic immune response syndrome (SIRS): when inflammatory mediators are released into the systemic circulation. Close collaboration with the anaesthetist is necessary.

The prognosis is strongly linked to the timing of surgery: ‘time is parenchyma’ [9]. A study has shown that at least 1/3 of thoracic surgeons have seen lobar torsion in their patients. This is a rare but classic condition [10].

## Conclusion

Herein, we describe an exceptional case of a spontaneous, sub-acute pulmonary torsion, successfully treated by urgent lobectomy and detorsion. Torsion is classically known by the thoracic surgeons, but rarely encountered by the other specialists.

In conclusion, as the case described is very rare, it should only be suspected when similar images are seen on CT scan or when antibiotics are not sufficient to treat pneumonia. Rapid surgical management is the rule, preserving the ‘pink lobes’ and resecting the necrotic lobes. Surgery is generally facilitated by the anatomy, and it is important to remember to clamp the pulmonary veins before reducing the torsion to avoid embolization of a clot.
